# Intraspecific variation in animal mating signals: a test of Mayr's conjecture

**DOI:** 10.1093/beheco/arag028

**Published:** 2026-03-16

**Authors:** David A Gray

**Affiliations:** Department of Biology, California State University Northridge, 18111 Nordhoff Street, Northridge, CA 91330-8303, United States

**Keywords:** mating signals, reproductive isolation, sexual selection, mate recognition

## Abstract

Mayr proposed that the stringency of stabilizing selection on animal mating signals is context-dependent: in species lacking sympatric congeners, relaxed selection pressure would afford greater intraspecific variability. This idea has rarely (if ever) been directly tested. Here, I evaluate Mayr's conjecture using a comprehensive dataset on acoustic mating signals from 39 taxa (33 named species and 6 unnamed lineages) of North American *Gryllus* field crickets. In doing so, I distinguish between 2 distinct functions of mating signal components: recognition versus persuasion. Contrary to Mayr's prediction, intraspecific variation in recognition mating signals did not increase in species with fewer or no sympatric congeners. Stabilizing selection on recognition traits appears to be maintained across both isolated and sympatric populations, possibly due to selection for efficient intraspecific communication—aligning with some aspects of Paterson's “Specific Mate Recognition System” model. Persuasion traits, on the other hand, show elevated levels of variation consistent with directional sexual selection promoting condition dependence. Together these results reveal the ubiquity of stabilizing selection on recognition traits when at evolutionary equilibrium, and the critical importance of distinguishing between recognition and persuasion functions of animal mating signals.

## Teaser

This paper presents 2 significant advances of interest to a broad audience of biologists engaged in research on speciation and the evolution of reproductive isolation, sexual selection, and/or mating signal evolution. The 2 significant advances are (i) testing for the first time a 60+ year old prediction from Ernst Mayr regarding levels of variation in animal mating signals, and (ii) demonstrating empirically the importance of making a key distinction between recognition mating signals and persuasion mating signals, which despite being ∼75 years old (attributable to Niko Tinbergen), has largely been absent from modern discussions of mating signal evolution. The empirical work is based on phylogenetically informed analysis of an entire clade, so the findings apply broadly.

## Introduction

When no other closely related species occur, all courtship signals can ‘afford’ to be general, nonspecific and variable.—Mayr 1963, p. 109.

Mayr fundamentally viewed animal mating signals as adaptations shaped by selection to limit interspecific crosses and associated negative heterosis (Mayr 1942, 1963). In Mayr's view, mating signals were thus under natural selection, not sexual selection. As such, Mayr thought that mating signals should be under stabilizing selection and exhibit minimal intraspecific variation. However, because he viewed mating signals as relational with respect to co-occurring closely related species, he considered the stringency of stabilizing selection on mating signals to be a function of the presence or absence of those other species, as can be seen in the quote above. In support of the statement, Mayr (1963) cited somewhat anecdotal reports of birds on Tenerife and the Azores, eg, Marler (1958). I have not been able to find subsequent direct tests of this idea in the literature, although the volume of published works on sexual selection, speciation, and signal, mate, or species recognition published over 60+ years makes it entirely possible that I have missed some. At minimum, it has not been a well explored idea, perhaps for a variety of reasons: (i) Mayr's focus on natural selection shaping pre-zygotic isolation in sympatry led to a focus in the 1970s speciation literature on the pattern of reproductive character displacement, which tended to focus on the average values of mating signals among populations, rather than levels of variation within populations (Blair 1974; Walker 1974a; Bell 1976; Wasserman and Koepfer 1977; Waage 1979); (ii) when the field of sexual selection exploded from the 1970s to the present, it was quickly established that many mating signal traits exhibit high levels of intraspecific variation (Andersson 1982; Ryan 1990; Cuervo and Møller 2001), rather than low levels. This likely led to a neglect of Mayr's ideas, at least within the evolutionary behavioral ecology literature. That is, the burgeoning sexual selection literature mostly ignored speciation and mate recognition, just as Mayr had mostly ignored sexual selection (Mayr 1942, 1963, but, eventually, see Mayr 1972).

Progress toward integration of the speciation and sexual selection research agendas was catalyzed by influential papers by Lande (1981), Ryan and Rand (1993), and West-Eberhard (1983). However, the apparent contradiction remained: the speciation expectation that mating signals should be subject to stabilizing selection and show low intraspecific variation, and the behavioral ecology empirical findings of directional sexual selection and high intraspecific variation. This contradiction is potentially resolved by realization that mating signals comprise multiple parts, sometimes temporally separated and sometimes in different modalities, in which “reproductive isolation” recognition functions and sexual selection “persuasion” functions (sensu Tinbergen 1953, p. 23) are accomplished via different signal traits or sets of signal traits which differ in their patterns of selection and expected variation (Verrell 1988; Gerhardt 1991; McPeek et al. 2008; Gray 2022). How that variation depends on the presence of heterospecifics remains largely unresolved.

Here I present data on intraspecific variation in cricket mating signals as a function of (i) the potential for gene flow from sympatric or parapatric congeners, and (ii) whether the signal components function as “recognition” traits or as “persuasion” traits. I do this via analysis of the acoustic mating signals and geographic ranges of the complete fauna of acoustically communicating *Gryllus* field crickets in North America north of Mexico, consisting of 33 named species plus 6 unnamed independent genetic lineages (Weissman and Gray 2019; Gray et al. 2020); 2 additional species which do not produce calling song were excluded from the analysis (*Gryllus ovisopis* and *Gryllus cayensis* [Walker 1974b, 2001; Gray et al. 2018]). The functions of different components of cricket songs are already well established: dominant frequency (FREQ), pulse rate (PRATE), and pulse duty cycle (PDC) are the critical “recognition” traits (Popov and Shuvalov 1977; Kostarakos and Hedwig 2012; Hennig et al. 2014; Schöneich et al. 2015; Gabel et al. 2016; Schöneich 2020; Clemens et al. 2021), known to be subject to stabilizing female responses (Blankers et al. 2015; Gray et al. 2016a; Hennig et al. 2016; Bailey et al. 2017; Gray 2022), whereas pulses per chirp, chirp rate, and chirp duty cycle are “persuasion” traits generally subject to directional female mate preferences favoring greater acoustic stimulus (Popov and Shuvalov 1977; Blankers et al. 2015; Hennig et al. 2016; Gray et al. 2016a; Bailey et al. 2017; Gray 2022). The mating signal traits are evaluated by the female cricket sensory-neural system in an order-of-operations manner (Hedwig 2016; Gray 2022) such that increased response to the persuasion traits is contingent upon species-specific values of the recognition traits.

## Materials and methods

### Song analysis

Details of the recording and analysis of cricket mating signals can be found in Weissman and Gray (2019) and Gray (2022). Briefly, as part of a major taxonomic revision of the genus (Weissman and Gray 2019), wild crickets (mostly located by ear), were captured in natural habitats and held in individual containers and then audio recorded when calling under laboratory conditions, mostly by D.B. Weissman. Males were thus of unknown age and dietary history; the variation reflecting the natural variation in the wild. This taxonomic fieldwork spanned several decades; cricket calls analyzed for this work were recorded between 1985 and 2017. Calls were selected for analysis to be those recorded at or near 25 °C. I analyzed songs of 5 wild-caught males per species using Audacity software (audacityteam.org); I measured 5 different exemplars for each song trait for each individual. Measurements were then averaged within individuals to generate individual means per song trait, and those individual means were then averaged to generate species means, standard deviations (SD), and coefficients of variation (species SD/mean) per song trait per species.

### Potential for gene flow

Based on Weissman and Gray (2019), for each species, I created a matrix of species co-occurrences, and then summed the number of sympatric congeners for each species. Sympatry was determined by actual co-occurrences, not just general overlap on range maps; range maps are nonetheless useful and for all species are available in Weissman and Gray (2019) and also available online (https://orthsoc.org/sina/cricklist.htm#gryllinae). Geographic ranges are assumed to be stable over the timeframe of the past few decades, although stability on the scale of millennia is unlikely (both past and future). I also created a less conservative index by repeating the analysis counting both sympatric and parapatric congeners (geographically and/or ecologically adjacent) as potential sources of gene flow (Larson et al. 2013; Gray et al. 2016b; Blankers et al. 2018; Talavera et al. 2021). Numbers of sympatric congeners ranged from 0 to 11 (5 species with zero); numbers of sympatric or parapatric congeners ranged from 1 to 20.

### Statistical analysis

All analyses were conducted in R 4.3.1. Phylogenetically informed analyses used a well resolved multilocus molecular phylogeny of *Gryllus* (Gray et al. 2020). For clarity, I analyzed the data for each trait separately using both phylogenetically naïve simple regression and phylogenetically informed independent contrasts calculated using the function “pic” in the “ape” package (Paradis et al. 2004). Additionally, I used a Bayesian modeling approach in the R package “MCMCglmm” (Hadfield 2010) to test the effect of recognition v. persuasion sets of traits within a phylogenetically controlled analysis framework (Hadfield 2015; Todorov et al. 2021). The model simultaneously controls for phylogeny, and tests for effects of numbers of sympatric congeners [*S*], type of trait (recognition versus persuasion) [TYPE], and the *S*:TYPE interaction, as follows:


$$CV_{ij}=\beta 0+\beta 1\times S+\beta 2\times \mathrm{TYPE}+\beta 3\times S:\mathrm{TYPE}+u_{i}+\varepsilon_{ij}$$

where *u_i_* ∼ *N*(0, $\sigma_{\mathrm{phylo}}^{2}$) is the phylogenetic random effect; *ε_ij_* ∼ *N*(0, $\sigma_{\mathrm{resid}}^{2}$) is the residual error.

The model used parameter-expanded inverse-gamma (0.002, 1) priors, (ie, flat/weakly informative), and ran for 13,000 iterations, burnin = 3,000, and thinning interval of 10; model diagnostics (effective sample sizes) validated their appropriateness.

A linear modeling approach may not be intuitive given Mayr's implied categorical contrast between taxa with zero sympatric congeners and those with one or more sympatric congeners. However, consideration of interspecific constraints on mating signal variation reveals that a linear approach is justified (see Fig. S1).

## Results

**Fig. 1.**
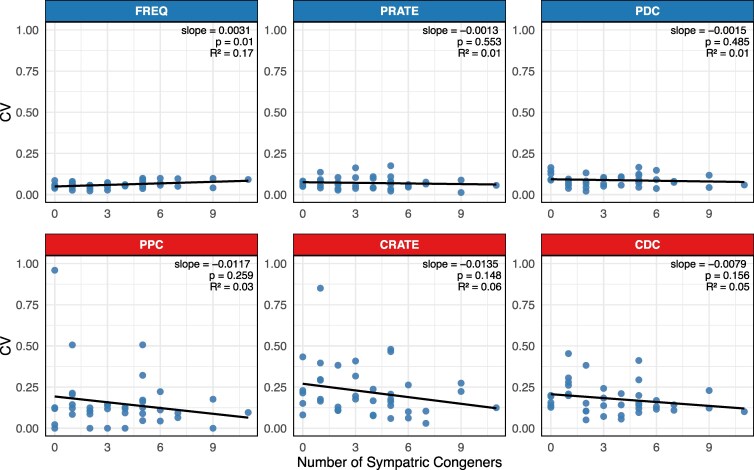
Relationship between intraspecific mating signal variation, measured as coefficient of variation, CV (SD/mean) and the number of sympatric congeners; for all comparisons, N = 39. The mating signal recognition traits (top panel, blue header) are dominant frequency (FREQ), pulse rate (PRATE), and pulse duty cycle (PDC); the mating signal persuasion traits (lower panel, red header) are pulses per chirp (PPC), chirp rate (CRATE), and chirp duty cycle (CDC).

Intraspecific variation in 2 of the mating signal “recognition” traits, PRATE and PDC, shows no relationship with the number of sympatric congeners; the third mating signal recognition trait, FREQ, actually shows a significantly positive relationship with the number of sympatric congeners, opposite Mayr's prediction (Fig. 1, top panel). None of the 3 mating signal “persuasion” traits shows a statistically significant relationship, although all 3 trend toward lower variation with more sympatric congeners (Fig. 1, lower panel). Similar results were obtained using both sympatric and parapatric congeners as potential sources of gene flow (Fig. S2) and when using phylogenetically independent contrasts (all *P* > 0.2, Figs S3 and S4).

**Fig. 2.**
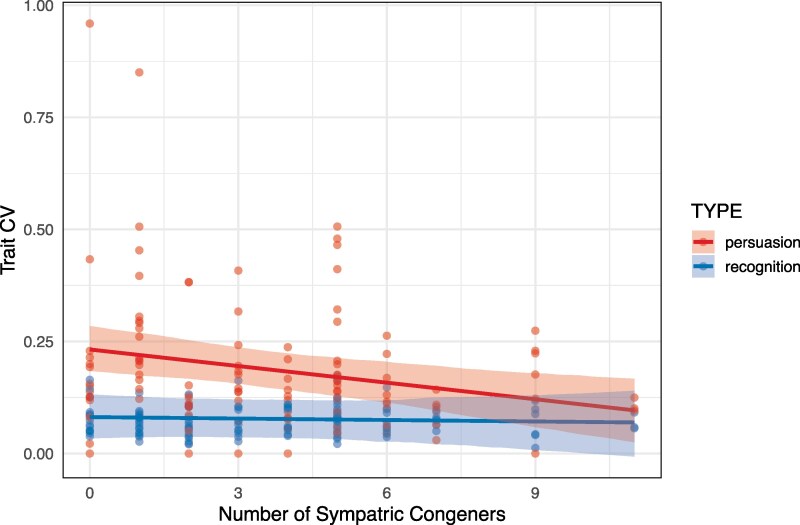
Relationship between CV and number of sympatric congeners by type of trait, recognition or persuasion, using phylogenetically informed modelling.

Phylogenetically controlled Bayesian models from “MCMCglmm” show the same results, but additionally directly test for an effect of trait TYPE (recognition versus persuasion) and the interaction between TYPE and number of sympatric congeners (Fig. 2, Table 1). The credible interval (CI) for the slope of the recognition traits includes zero (mean −0.0011, *l*-95% −0.0087, *u*-95% 0.0070), whereas the CI for the slope of the persuasion traits does not (mean −0.0122, *l*-95% −0.0207, *u*-95% −0.0046). Considering both sympatric and parapatric congeners gives the same overall interpretation (Fig. S5 and Table S1).

**Table 1:** Results of a Bayesian “MCMCglmm” model with random effect reflecting phylogenetic history, location effects of number of sympatric taxa [*S*], type of traits, recognition or persuasion [TYPE, with persuasion as the reference level], and the *S*:TYPE interaction.

	Posterior mean	*l*-95% CI	*u*-95% CI	Effective sample	pMCMC
(Intercept)	0.2319	0.1851	0.2804	1,000	<0.001
*S*	−0.0122	−0.0207	−0.0046	1,000	0.002
TYPErecognition	−0.1500	−0.1909	−0.1107	1,000	<0.001
*S*:TYPErecognition	0.0111	0.0025	0.0213	1,000	0.024
∼Phylogeny	0.002261	0.0004865	0.004312	818.4	…
∼Residual	0.009755	0.007907	0.01192	1,000	…

## Discussion

These results are novel and interesting for 2 reasons: (i) they do not support the idea of increased intraspecific mating signal variation for recognition traits in depauperate faunas (contra Mayr), (ii) they further support the importance of distinguishing between recognition and persuasion functions when considering the evolutionary trajectories of different components of animal mating signals (Tinbergen 1953; Verrell 1988; Gray 2022). I discuss each in turn.

Mayr's conjecture regarding the stringency of stabilizing selection reflects his understanding of animal mating signals as adaptations evolved because of their benefit in keeping gene pools distinct; ie, they are isolating mechanisms selected to function in reducing gene flow from congeners (Mayr 1963). As pointed out by Paterson (1993), this is an inherently relational view of animal mating signals. Paterson instead advocated the view that recognition signals promoted intraspecific efficiency of pairing, what he called a “Specific Mate Recognition System,” subjected to stabilizing selection independent of the presence or absence of other species. Indeed, Paterson explicitly rejected the idea of reinforcement. In many respects, the field has moved on: Paterson's recognition concept of species has been widely rejected (Coyne et al. 1988), and there are now numerous examples supporting the idea that the presence of sympatric congeners does indeed shape mating signal properties, in particular the average values of mating signal components, recently reviewed in Shaw et al. (2024). For example, reproductive character displacement, in which the average values of mating signals are more divergent in sympatry than in allopatry, has been found in a number of cases (Marshall and Cooley 2000; Lemmon 2009; Yukilevich 2021), as has the complimentary pattern of character displacement in female recognition of male signal traits (Höbel and Gerhardt 2003) and/or stringency of female preference (Tinghitella and Zuk 2009; Symes 2014). A related pattern of faster and more complete interspecific divergence in mating signals and/or prezygotic reproductive isolation in sympatric taxa (Coyne and Orr 1989, 1997; Matute and Cooper 2021; Yukilevich 2023; Gray et al. unpublished) also supports the idea that the presence of congeners shapes the evolutionary trajectories of mating signal traits. So why then is Mayr's prediction not met?—logically, absent constraints from sympatric congeners, mating signals would indeed be free from this form of stabilizing selection as there is no risk of hybridization. Perhaps the explanation partially vindicates Paterson: stabilizing selection for efficiency of intraspecific pairing could constrain variation in mating signal traits in isolation despite relaxed selection given absence of congeners. We can reconcile these theoretical views, as well as the empirical evidence showing effects of sympatry, by restating Mayr's conjecture: the presence of closely related heterospecifics causes divergent selection on mate recognition signals sufficient to overcome the stabilizing selection normally in effect due to efficiency of intraspecific pairing; once at equilibrium, stabilizing selection for efficient intraspecific pairing returns and maintains minimal intraspecific variation regardless of the presence or absence of closely related heterospecifics.

The above discussion has been in reference to recognition signals only. The other contribution of this work is to provide further evidence that the study of mating signals, whether primarily focused on reproductive isolation and speciation or on sexual selection and mate choice, needs to distinguish between recognition and persuasion functions of mating signals. The distinction was made almost 75 years ago (Tinbergen 1953), but seems to have been mostly lost (Candolin 2003; Wilkins et al. 2013; Schaefer and Ruxton 2015; Mitoyen et al. 2019), notable exceptions include (Verrell 1988; Hebets and Papaj 2005; McPeek et al. 2008; Arbuthnott et al. 2010). To be unambiguously associated with conspecifics, recognition traits, which are under stabilizing selection at equilibrium (due to either interspecific interactions [Mayr's view] or intraspecific efficiency of pairing [Paterson's view], or both [my view]), should be stereotyped with low intraspecific variation; persuasion traits, which are typically under directional sexual selection (Ryan and Keddy-Hector 1992; Andersson and Simmons 2006), maintain their persuasive value because they are high-effort displays (Byers et al. 2010; Manica et al. 2016), which thereby reveal variation in phenotypic quality (Rowe and Houle 1996; Brandt and Greenfield 2004; Dugand et al. 2019; Baur and Berger 2020; Ferreira and Lüpold 2021; Parrett et al. 2022; Sly et al. 2022; Valiya Parambil and Isvaran 2025). The different functions select for different design properties, therefore it seems unlikely that any single mating signal component can simultaneously be optimized for both functions.

How can researchers distinguish between recognition and persuasion functions? There is likely no single universal property, but consideration of 5 features is likely to provide significant insight. (i) Levels of intraspecific variation: recognition traits should have low intraspecific variation whereas persuasion traits will usually have higher levels of intraspecific variation. (ii) Phenotypic plasticity: recognition traits should be relatively robust to perturbation during development, whereas persuasion traits may develop condition-dependent trait expression in all but the most benign developmental environments (Rowe and Houle 1996). (iii) Temporal or neural priority: because the fitness consequences of conspecific versus heterospecific mating will usually exceed the fitness consequences of mate selection within a pool of recognized conspecifics (Gray 2022), we would expect recognition traits to have priority over persuasion traits. This may often take the form of temporal priority, such that recognition signals are produced earlier in a mating sequence than persuasion signal traits. For example, I would predict that bird calls which from a distance advertise the location of a lek are likely to contain predominantly stereotyped recognition features, whereas displays given on the lek may contain both recognition and persuasion features. Sensory-neural priority is a non-exclusive alternative to temporal priority: sensory-neural mating signal recognition may often be both necessary and sufficient to enable mating, whereas the stimulus value of persuasion traits is likely conditional on activation of recognition circuits—to cite a well-known example, in túngara frogs “whines” are categorical recognition traits (Baugh et al. 2008) but “chucks” enhance the attractiveness of “whines” (Ryan 1985). (iv) Mate preference function shape: recognition traits are “recognized” by closed preference functions acting as band-pass filters, whereas persuasion traits are typically subject to directional preference functions (Rodríguez et al. 2013; Kilmer et al. 2017); in *Gryllus* field crickets detailed female preference function analyses support these generalizations (reviewed in Gray 2022). (v) Phylogenetic signal: across a clade, recognition traits are likely to change in arbitrary directions during speciation, eroding phylogenetic signal, whereas persuasion traits may show continuity of signaling value, to some degree enabling build-up of phylogenetic autocorrelation. One important consideration is that researchers need not necessarily identify different traits in order to distinguish recognition and persuasion functions—the “qualities” of a trait could promote recognition (eg, the presence of specific elements of a bird's song), whereas the display effort or courtship performance value (sensu Hebets et al. 2024) of that same trait could promote persuasion (eg, the bird singing those specific song elements over and over for hours on a cold morning) (Nishida and Takagi 2018; Gil and Llusia 2020).

One interesting, and unexpected, result was the negative relationship between persuasion mating signal variation and the number of sympatric congeners. For Mayr's conjecture, the relevant result is the absence of such a relationship in the recognition traits, but why does it appear to be the case that there is such a relationship in the persuasion traits, especially when taken collectively (Fig. 2)? Clearly this is not predicted by Mayr, Paterson, or the reconciled Mayr–Paterson view suggested above, as those ideas specifically apply only to recognition traits. Increased intraspecific variation in persuasion traits suggests heightened condition dependent expression. Perhaps species living in species-rich faunas are subject to greater niche-partitioning due to interspecific competition, and are thus more specialized, leading to greater compatibility between local genotype and local environment—thus reducing condition dependent genotype × environment effects, and so lower coefficient of variation (CV). In contrast, species living alone can occupy a wider ecological niche space, but as a result are less locally adapted to any particular local environmental variation—thus increasing condition dependent genotype × environment effects, and so higher CV. At present, there is no evidence in support of this idea, although abundant evidence does suggest that G × E interactions enhance total variation from condition dependent expression of sexual signals (David et al. 2000; Jia et al. 2000).

Limitations of the current approach: despite these data being the most comprehensive test of Mayr's conjecture to date, they are not without limitations and caveats. First, using 5 individuals per species to calculate species level CV is a fairly small sample size. The accuracy of the CV calculations could surely be improved with additional samples analyzed—but the error is expected to be random and in no way expected to be systematically related to sympatry, or to differ between recognition and persuasion traits. The 5 individuals were selected so as to minimize variation in recording temperature; additional samples recorded at or close to 25 °C are not available for all 39 species. Second, potential geographic variation in mating signals and in species' geographic range overlap is not explicitly considered in the analysis. That is, the present analysis treats variation and levels of sympatry as species level traits, however different populations could vary in their mating signals and/or in numbers or degree of geographic overlap with congeners. This is likely to cause some error in estimation of the potential for gene flow from sympatric congeners when treated as a species level trait. However, that error is expected to be random with respect to estimated mating signal CV, and would not differ for recognition and persuasion traits. That is, the empirical limitations of the current data are unlikely to bias the overall results, would not cause a systematic difference between recognition and persuasion traits, and are offset by the analysis consisting of the complete clade of all 39 acoustically signaling North American *Gryllus* species.

In summary, despite their limitations, these data support 2 main conclusions: (i) mate recognition mating signals and mate persuasion mating signals cannot be treated together; their distinct functions dictate different properties and different evolutionary trajectories. (ii) Mate recognition mating signals will at equilibrium be under stabilizing selection from both efficiency of intraspecific pairing and, at equilibrium in sympatry, distinctness from heterospecific signals. From this it follows that speciation will involve relatively rapid changes in mate recognition signals (and preferences), followed by stasis (Lande 1981; Iwasa and Pomiankowski 1995), whereas divergent populations or species would nonetheless be characterized by persistent directional sexual selection on persuasion signal traits (McPeek et al. 2008; Arbuthnott et al. 2010). This way of thinking also renders moot the nearly endless discussion of whether mating signals are arbitrary traits or condition-dependent indicators of phenotypic quality (Johnstone 1995; Jones and Ratterman 2009; Schaefer and Ruxton 2015; Számadó et al. 2023): in my view, recognition traits are likely to be arbitrary and evolve via the Fisher–Lande or sensory drive models (Boughman 2002; Arnold and Houck 2016), whereas persuasion traits are likely to evolve via the indicator model (Rowe and Houle 1996; Lorch et al. 2003).

## Supplementary Material

arag028_Supplementary_Data

## Data Availability

Analyses reported in this article can be reproduced using the data provided by Gray (2026).
