# Supplementary material for: Intraspecific variation in animal mating signals: a test of Mayr's conjecture
Source: Behav Ecol. 2026 Mar 16;37(3):arag028. doi: 10.1093/beheco/arag028 (PMC13017002; doi:10.1093/beheco/arag028)
Supplement: arag028_Supplementary_Data [file arag028_supplementary_data.docx]

README: Supplementary materials includes two parts, Supplemental Figures and Tables, and Data and Code

Intraspecific variation in animal mating signals: a test of Mayr’s conjecture: Supplemental Figures and Tables

Figure S1. Conceptual plot of the interspecific constraints on phenotypic variation: top panel shows from 1 to 6 species within a shared range, with ~ indicating a direction of unconstrained phenotypic variation and T indicating a direction of constrained phenotypic variation; bottom panel shows the proportion of the taxa able to vary in at least one direction – approximating a linear trend.

Figure S2. Relationship between intraspecific mating signal variation, measured as coefficient of variation, CV (SD/mean) and the number of sympatric or parapatric congeners; for all comparisons, N = 39. The mating signal recognition traits are dominant frequency (FREQ), pulse rate (PRATE), and pulse duty cycle (PDC); the mating signal persuasion traits are pulses per chirp (PPC), chirp rate (CRATE), and chirp duty cycle (CDC). Note that no species have zero sympatric or parapatric congeners.

Figure S3. Relationship between phylogenetically independent contrasts in intraspecific mating signal variation and phylogenetically independent contrasts in the number of sympatric congeners. For all comparisons, N = 38; traits as in Figs 1 and S2.

Figure S4. Relationship between phylogenetically independent contrasts in intraspecific mating signal variation and phylogenetically independent contrasts in the number of sympatric or parapatric congeners. For all comparisons, N = 38; traits as in Figs 1 and S2.

Figure S5. Relationship between CV and number of sympatric or parapatric congeners by type of trait, recognition or persuasion, using phylogenetically informed modelling.

Table S1. Results of a Bayesian MCMCglmm model with random effect G-structure reflecting phylogenetic history, location effects of number of sympatric or parapatric taxa [SNP], type of traits, recognition or persuasion [TYPE, with persuasion as the reference level], and the SNP:TYPE interaction.

|  | Posterior Mean | l-95% CI | u-95% CI | Effective Sample | pMCMC |
| --- | --- | --- | --- | --- | --- |
| (Intercept) | 0.2471 | 0.2003 | 0.2982 | 1000 | <0.001 |
| SNP | -0.0076 | -0.0117 | -0.0039 | 1093.1 | 0.001 |
| TYPErecognition | -0.1639 | -0.2037 | -0.1188 | 1000 | <0.001 |
| SNP:TYPErecognition | 0.0070 | 0.0022 | 0.0116 | 913.2 | <0.001 |
|  |  |  |  |  |  |
| G-structure: ~Phylogeny | 0.0021 | 0.0004 | 0.0042 | 832.5 |  |
| R-structure: ~units | 0.0096 | 0.0077 | 0.0116 | 899 |  |

SNP recognition trait slope: mean -0.0008, l-95% -0.0049, u-95% 0.0032

SNP persuasion trait slope: mean -0.0077, l-95% -0.0119, u-95% -0.0035

Intraspecific variation in animal mating signals: a test of Mayr’s conjecture: Supplemental Data and Code

**Data**: Species, SNP [=number of sympatric and/or parapatric taxa], S [=number of Sympatric taxa], CV for traits FREQ, PRATE, PDC, PPC, CRATE, CDC

| Taxon | SNP | S | FREQ | PRATE | PDC | PPC | CRATE | CDC |
| --- | --- | --- | --- | --- | --- | --- | --- | --- |
| G_brevicaudus | 5 | 3 | 0.07197672 | 0.16263125 | 0.1046474 | 0 | 0.19583422 | 0.0714087 |
| G_thinos | 1 | 1 | 0.04481136 | 0.07861139 | 0.08687512 | 0.08408643 | 0.39624444 | 0.45330943 |
| G_veintinueve | 8 | 3 | 0.02726741 | 0.05341279 | 0.05092651 | 0.1381927 | 0.31682361 | 0.24198968 |
| G_assimilis | 9 | 4 | 0.05669662 | 0.03937905 | 0.05716627 | 0.11907012 | 0.1668333 | 0.07781205 |
| G_multipulsator | 10 | 5 | 0.03637703 | 0.10880735 | 0.0914432 | 0.32142857 | 0.20686911 | 0.0951454 |
| G_firmus_FL | 3 | 3 | 0.07288445 | 0.10341029 | 0.06457679 | 0.1177898 | 0.40808752 | 0.18358489 |
| G_pennsylvanicus | 10 | 3 | 0.04579527 | 0.0373778 | 0.09789484 | 0.14541774 | 0.17659481 | 0.13740903 |
| G_firmus_TX | 5 | 2 | 0.02229878 | 0.06072632 | 0.02070167 | 0 | 0.10815489 | 0.10419714 |
| G_rubens | 9 | 4 | 0.05181768 | 0.10915111 | 0.10879443 | 0.0931695 | 0.07571167 | 0.05576648 |
| G_regularis | 9 | 6 | 0.07015284 | 0.05158635 | 0.14752311 | 0.04384447 | 0.10039008 | 0.11549608 |
| G_texensis | 12 | 6 | 0.05683493 | 0.04539066 | 0.0909995 | 0.22219509 | 0.26279486 | 0.13091012 |
| G_integer | 13 | 9 | 0.09864894 | 0.08820452 | 0.11776 | 0.17634594 | 0.27396086 | 0.12241743 |
| G_armatus | 19 | 11 | 0.0915766 | 0.05626551 | 0.05862468 | 0.09581306 | 0.1247262 | 0.10079076 |
| G_vernalis | 4 | 2 | 0.03818752 | 0.10420566 | 0.04390593 | 0.10480424 | 0.11189108 | 0.15195855 |
| G_fultoni | 5 | 4 | 0.06083899 | 0.0423243 | 0.1030308 | 0.1281096 | 0.23730269 | 0.21033995 |
| G_planeta | 3 | 2 | 0.05503442 | 0.03548425 | 0.08247183 | 0.08600261 | 0.12918419 | 0.10422071 |
| G_montis_Clade_1 | 2 | 1 | 0.04681136 | 0.08179059 | 0.06872514 | 0.12184369 | 0.17592321 | 0.30537012 |
| G_veletis | 20 | 9 | 0.04106497 | 0.01257592 | 0.04316617 | 0 | 0.22344691 | 0.22909772 |
| G_cohni | 7 | 5 | 0.09910294 | 0.1752266 | 0.11691654 | 0.125491 | 0.46542986 | 0.41129523 |
| G_vocalis | 14 | 7 | 0.05024282 | 0.07599672 | 0.07420986 | 0.0925734 | 0.02984887 | 0.10899592 |
| G_personatus | 13 | 5 | 0.09013371 | 0.07980001 | 0.08133999 | 0.1711254 | 0.13793489 | 0.29404142 |
| G_staccato | 10 | 5 | 0.05523342 | 0.06264623 | 0.0443471 | 0.08887668 | 0.16243031 | 0.12063974 |
| G_lineaticeps | 8 | 5 | 0.08370335 | 0.03272469 | 0.16600627 | 0.16109487 | 0.05930859 | 0.19911815 |
| G_chisosensis | 2 | 1 | 0.08024958 | 0.13583335 | 0.08538199 | 0.50601516 | 0.85027298 | 0.27939967 |
| G_veletisoides | 9 | 5 | 0.0478094 | 0.07803414 | 0.07039504 | 0.50640879 | 0.47932998 | 0.15195053 |
| G_montis_Clade_3 | 6 | 0 | 0.05939762 | 0.0736536 | 0.12616187 | 0.1250857 | 0.22897215 | 0.13806768 |
| G_montis_Clade_2 | 4 | 0 | 0.08550461 | 0.04974646 | 0.09203171 | 0.11907012 | 0.15089891 | 0.19956991 |
| G_transpecos | 8 | 5 | 0.07296393 | 0.02138439 | 0.12408952 | 0.04610449 | 0.17578927 | 0.14016275 |
| G_lightfooti | 15 | 7 | 0.09664168 | 0.06391304 | 0.08053972 | 0.06446026 | 0.10404091 | 0.14254017 |
| G_sotol | 2 | 1 | 0.02650969 | 0.09163388 | 0.09482659 | 0.21323031 | 0.29147812 | 0.20811834 |
| G_vulcanus | 3 | 0 | 0.04529393 | 0.05079036 | 0.08781443 | 0.95927778 | 0.43330096 | 0.15588654 |
| G_longicercus | 17 | 6 | 0.09900651 | 0.06121037 | 0.03701448 | 0.11106107 | 0.06184502 | 0.16867023 |
| G_montis_Clade_4 | 1 | 1 | 0.07304051 | 0.04025334 | 0.05671378 | 0.14369782 | 0.29607857 | 0.19741595 |
| G_navajo | 3 | 1 | 0.06262975 | 0.05501361 | 0.03770868 | 0.20548047 | 0.16426931 | 0.26060156 |
| G_leei | 2 | 0 | 0.05115735 | 0.0641768 | 0.14356634 | 0.02213929 | 0.21438717 | 0.19292564 |
| G_saxatilis_Tulare | 4 | 2 | 0.05741226 | 0.07306891 | 0.13192966 | 0.1244824 | 0.38245573 | 0.3819888 |
| G_saxatilis_Mormoni | 4 | 0 | 0.03824203 | 0.08234003 | 0.16446507 | 0 | 0.08106319 | 0.12682684 |
| G_saxatilis | 13 | 4 | 0.05616117 | 0.09789401 | 0.10201871 | 0 | 0.07998331 | 0.14171982 |
| G_makhosica | 2 | 2 | 0.05134084 | 0.02701744 | 0.06727782 | 0.10647943 | 0.10756557 | 0.05079822 |

**CODE:**

#### NOTE: MCMCglmm runs in two separate code blocks to clear the environment; it is important to re-start a new R session between code blocks.

##BLOCK 1:

# === Load packages ===

library(ape)

library(phytools)

library(dplyr)

library(tidyr)

library(MCMCglmm)

# === Step 1: Load raw data and tree ===

tree <- read.nexus("GryllusbestUS.nex")

data <- read.csv("complexity_data.csv", header = TRUE, row.names = 1)

data$Species <- rownames(data)

# === Step 2: Define trait types ===

recognition_traits <- c("FREQ", "PRATE", "PDC")

persuasion_traits <- c("PPC", "CRATE", "CDC")

# === Step 3: Create long-format data with TYPE ===

long_pgls <- data %>%

pivot_longer(cols = c(FREQ, PRATE, PDC, PPC, CRATE, CDC),

names_to = "Metric", values_to = "CV") %>%

mutate(

TYPE = case_when(

Metric %in% recognition_traits ~ "recognition",

Metric %in% persuasion_traits ~ "persuasion"

),

TYPE = factor(TYPE),

Metric = factor(Metric)

)

# === Step 4: Prepare model data for S predictor ===

species_levels <- sort(unique(as.character(long_pgls$Species)))

tree_mcmc <- drop.tip(tree, setdiff(tree$tip.label, species_levels))

tree_mcmc$tip.label <- sort(tree_mcmc$tip.label)

tree_mcmc <- force.ultrametric(tree_mcmc, method = "extend")

tree_mcmc$node.label <- NULL

data_mcmc <- long_pgls %>%

filter(Species %in% tree_mcmc$tip.label, !is.na(CV), !is.na(S), !is.na(TYPE)) %>%

mutate(

animal = as.character(Species),

TYPE = factor(TYPE)

)

invA <- inverseA(tree_mcmc, nodes = "TIPS")$Ainv

species_order <- sort(unique(data_mcmc$animal))

invA <- invA[species_order, species_order]

# === Step 5: Save for clean modeling in new session ===

saveRDS(data_mcmc, "data_mcmc_S.rds")

saveRDS(invA, "invA_S.rds")

####Code Block 2: Run MCMCglmm + Plot (Start in a fresh R session)

# === Clean environment ===

rm(list = ls())

gc()

# === Load packages ===

library(MCMCglmm)

library(dplyr)

library(tibble)

library(ggplot2)

# === Load saved RDS files ===

data_mcmc <- readRDS("data_mcmc_S.rds")

invA <- readRDS("invA_S.rds")

# === Force correct ordering of invA ===

species_order <- sort(unique(data_mcmc$animal))

invA <- invA[species_order, species_order]

# === Force character matching (not factor) ===

data_mcmc$animal <- as.character(data_mcmc$animal)

# === Final identity check ===

identical(species_order, rownames(invA)) # should return TRUE

saveRDS(data_mcmc, "data_mcmc_S_aligned.rds")

saveRDS(invA, "invA_S_aligned.rds")

# Clean environment

rm(list = ls())

gc()

# Load cleaned, aligned objects

data_mcmc <- readRDS("data_mcmc_S_aligned.rds")

invA <- readRDS("invA_S_aligned.rds")

# === Set priors ===

priors <- list(

G = list(G1 = list(V = 1, nu = 0.002)),

R = list(V = 1, nu = 0.002)

)

# === Fit the model ===

model_S <- MCMCglmm(

CV ~ S * TYPE,

random = ~ animal,

family = "gaussian",

ginverse = list(animal = invA),

data = data_mcmc,

prior = priors,

nitt = 13000, burnin = 3000, thin = 10

)

summary(model_S)

# === Extract posterior lines ===

post <- model_S$Sol

S_range <- seq(min(data_mcmc$S), max(data_mcmc$S), length.out = 100)

get_lines <- function(type) {

intercept <- post[, "(Intercept)"]

slope <- post[, "S"]

if (type == "recognition") {

intercept <- intercept + post[, "TYPErecognition"]

slope <- slope + post[, "S:TYPErecognition"]

}

preds <- outer(slope, S_range, `*`) + intercept

tibble(

S = S_range,

TYPE = type,

mean = apply(preds, 2, mean),

lower = apply(preds, 2, quantile, 0.025),

upper = apply(preds, 2, quantile, 0.975)

)

}

lines_recognition <- get_lines("recognition")

lines_persuasion <- get_lines("persuasion")

plot_data <- bind_rows(lines_recognition, lines_persuasion)

# === Plot ===

ggplot(data_mcmc, aes(x = S, y = CV, color = TYPE)) +

geom_point(alpha = 0.5, size = 2) +

geom_line(data = plot_data, aes(x = S, y = mean, color = TYPE), size = 1.2) +

geom_ribbon(data = plot_data, aes(x = S, ymin = lower, ymax = upper, fill = TYPE),

alpha = 0.25, color = NA, inherit.aes = FALSE) +

scale_color_manual(values = c("recognition" = "#1f78b4", "persuasion" = "#e31a1c")) +

scale_fill_manual(values = c("recognition" = "#1f78b4", "persuasion" = "#e31a1c")) +

labs(

x = "Number of Sympatric Congeners",

y = "Trait CV",

color = "TYPE", fill = "TYPE"

) +

theme_minimal(base_size = 14) +

theme(panel.border = element_rect(color = "black", fill = NA))

###export slope estimates and credible intervals

post <- model_S$Sol

# Slope for persuasion (reference level)

posterior_S_persuasion <- post[, "S"]

# Slope for recognition (add interaction term)

posterior_S_recognition <- post[, "S"] + post[, "S:TYPErecognition"]

summary_df <- tibble(

TYPE = c("persuasion", "recognition"),

slope_mean = c(mean(posterior_S_persuasion), mean(posterior_S_recognition)),

slope_median = c(median(posterior_S_persuasion), median(posterior_S_recognition)),

CI_lower = c(quantile(posterior_S_persuasion, 0.025), quantile(posterior_S_recognition, 0.025)),

CI_upper = c(quantile(posterior_S_persuasion, 0.975), quantile(posterior_S_recognition, 0.975))

)

print(summary_df)

####repeat same two code blocks for SNP analyses

#########Univariate analyses and Phylogenetic Independent Contrasts

# Clear the working environment

rm(list = ls())

setwd("~/……*your directory*")

mydata <- read.csv("complexity_data.csv", header = TRUE, sep = ",")

# Load required packages

if (!require("ggplot2")) install.packages("ggplot2", dependencies = TRUE)

if (!require("tidyr")) install.packages("tidyr", dependencies = TRUE)

if (!require("dplyr")) install.packages("dplyr", dependencies = TRUE)

if (!require("pwr")) install.packages("pwr", dependencies = TRUE)

library(ggplot2)

library(tidyr)

library(dplyr)

library(pwr)

# Reshape data to long format

long_data <- mydata %>%

pivot_longer(cols = c(FREQ, PRATE, PDC, PPC, CRATE, CDC),

names_to = "Metric", values_to = "Value")

# Set custom facet order

custom_order <- c("FREQ", "PRATE", "PDC", "PPC", "CRATE", "CDC")

long_data$Metric <- factor(long_data$Metric, levels = custom_order)

# Define custom y-limits

ylims <- data.frame(

Metric = custom_order,

ymin = rep(0, 6),

ymax = rep(1, 6)

)

# Join y-limits to long_data

long_data <- long_data %>%

left_join(ylims, by = "Metric")

# Reapply factor levels

long_data$Metric <- factor(long_data$Metric, levels = custom_order)

####FOR SYMPATRIC AND PARAPATRIC COMBINED

regression_data_snp <- long_data %>%

group_by(Metric) %>%

summarize(

model = list(lm(Value ~ SNP, data = cur_data())),

.groups = "drop"

) %>%

mutate(

coef = sapply(model, function(m) coef(m)[["SNP"]]),

p = sapply(model, function(m) summary(m)$coefficients["SNP", "Pr(>|t|)"]),

r2 = sapply(model, function(m) summary(m)$r.squared),

label_b = paste0("slope = ", round(coef, 4)),

label_p = paste0("p = ", ifelse(p < 0.001, "< 0.001", round(p, 3))),

label_r2 = paste0("R² = ", round(r2, 2))

)

ggplot(long_data, aes(x = SNP, y = Value)) +

geom_point(color = "steelblue") +

geom_smooth(method = "lm", se = FALSE, color = "black") +

geom_blank(aes(y = ymin)) +

geom_blank(aes(y = ymax)) +

facet_wrap(~ Metric, nrow = 2, scales = "free_y") +

geom_text(data = regression_data_snp, aes(x = Inf, y = Inf, label = label_b),

hjust = 1.1, vjust = 1.5, inherit.aes = FALSE, size = 3.2) +

geom_text(data = regression_data_snp, aes(x = Inf, y = Inf, label = label_p),

hjust = 1.1, vjust = 3, inherit.aes = FALSE, size = 3.2) +

geom_text(data = regression_data_snp, aes(x = Inf, y = Inf, label = label_r2),

hjust = 1.1, vjust = 4.5, inherit.aes = FALSE, size = 3.2) +

labs(x = "Number of Sympatric or Parapatric Congeners", y = "CV") +

theme_minimal(base_size = 12) +

theme(

panel.border = element_rect(color = "black", fill = NA, size = 0.8),

strip.background = element_rect(fill = "lightgray", color = "black"),

strip.text = element_text(face = "bold")

)

## FOR SYMPATRIC

regression_data_s <- long_data %>%

group_by(Metric) %>%

summarize(

model = list(lm(Value ~ S, data = cur_data())),

.groups = "drop"

) %>%

mutate(

coef = sapply(model, function(m) coef(m)[["S"]]),

p = sapply(model, function(m) summary(m)$coefficients["S", "Pr(>|t|)"]),

r2 = sapply(model, function(m) summary(m)$r.squared),

label_b = paste0("slope = ", round(coef, 4)),

label_p = paste0("p = ", ifelse(p < 0.001, "< 0.001", round(p, 3))),

label_r2 = paste0("R² = ", round(r2, 2))

)

ggplot(long_data, aes(x = S, y = Value)) +

geom_point(color = "steelblue") +

geom_smooth(method = "lm", se = FALSE, color = "black") +

geom_blank(aes(y = ymin)) +

geom_blank(aes(y = ymax)) +

facet_wrap(~ Metric, nrow = 2, scales = "free_y") +

geom_text(data = regression_data_s, aes(x = Inf, y = Inf, label = label_b),

hjust = 1.1, vjust = 1.5, inherit.aes = FALSE, size = 3.2) +

geom_text(data = regression_data_s, aes(x = Inf, y = Inf, label = label_p),

hjust = 1.1, vjust = 3, inherit.aes = FALSE, size = 3.2) +

geom_text(data = regression_data_s, aes(x = Inf, y = Inf, label = label_r2),

hjust = 1.1, vjust = 4.5, inherit.aes = FALSE, size = 3.2) +

labs(x = "Number of Sympatric Congeners", y = "CV") +

theme_minimal(base_size = 12) +

theme(

panel.border = element_rect(color = "black", fill = NA, size = 0.8),

strip.background = element_rect(fill = "lightgray", color = "black"),

strip.text = element_text(face = "bold")

)

##########################################################################

##########################################################################

################### phylogenetic correctness

if (!require("ape")) install.packages("ape")

if (!require("phytools")) install.packages("phytools")

if (!require("phylosignal")) install.packages("phylosignal")

if (!require("phylobase")) install.packages("phylobase")

if (!require("caper")) install.packages("caper")

library(ape)

library(phytools)

library(phylosignal)

library(phylobase)

library(caper)

tree <- read.nexus("~/…*filepath..*.GryllusbestUS.nex")

tree <- drop.tip(tree,c("G_ovisopis", "G_cayensis"))

plotTree(tree)

data <- read.csv("complexity_data.csv", header = TRUE, sep = ",", row.names = 1)

# Make sure species names in data match tree tip labels

# If needed: drop unmatched taxa

common_taxa <- intersect(rownames(data), tree$tip.label)

tree <- drop.tip(tree, setdiff(tree$tip.label, common_taxa))

data <- data[common_taxa, ]

##############re-do first analyses with phylogenetically independent contrasts

# Requires: dplyr, tidyr, ggplot2

# (optional but handy) broom

library(dplyr)

library(tidyr)

library(ggplot2)

####generate and save phylogenetic independent contrasts

library(ape)

library(tidyr)

library(dplyr)

# Ensure the tree has no node labels (avoid conflict with pic output)

tree$node.label <- NULL

# List of all traits to compute contrasts for

traits <- c("SNP", "S", "FREQ", "PRATE", "PDC", "PPC", "CRATE", "CDC")

# Ensure trait_data is a clean data frame with correct rownames

trait_data <- as.data.frame(trait_data) # if not already

trait_data <- trait_data[tree$tip.label, ] # reorder to match tree

# Compute PICs for each trait

pic_list <- lapply(traits, function(trait) {

x <- trait_data[[trait]]

names(x) <- rownames(trait_data)

pic(x, tree)

})

names(pic_list) <- traits

# Convert PIC list to a data frame

pic_data <- as.data.frame(pic_list)

# Add node IDs as rownames

pic_data$Node <- rownames(pic_list[[1]])

rownames(pic_data) <- pic_data$Node

# Final result: each row is an internal node; columns are PICs of each trait

head(pic_data)

# === 4. PIC(SNP) vs trait PICs — regression through origin ===

trait_subset <- c("FREQ", "PRATE", "PDC", "PPC", "CRATE", "CDC")

facet_order <- trait_subset

plot_data_snp <- pic_data %>%

pivot_longer(cols = all_of(facet_order),

names_to = "Metric", values_to = "Trait_PIC") %>%

mutate(SNP_PIC = rep(pic_data$SNP, times = length(facet_order)),

Metric = factor(Metric, levels = facet_order))

# per-panel slope/p, R² from lm(Trait_PIC ~ 0 + SNP_PIC)

reg_data_snp <- plot_data_snp %>%

group_by(Metric) %>%

summarize(

slope = coef(lm(Trait_PIC ~ 0 + SNP_PIC))[1],

p = summary(lm(Trait_PIC ~ 0 + SNP_PIC))$coefficients[1, 4],

r2 = summary(lm(Trait_PIC ~ 0 + SNP_PIC))$r.squared,

.groups = "drop"

) %>%

mutate(

label_slope = paste0("slope = ", round(slope, 3)),

label_p = paste0("p = ", ifelse(p < 0.001, "< 0.001", round(p, 3))),

label_r2 = paste0("R² = ", round(r2, 3))

)

ggplot(plot_data_snp, aes(x = SNP_PIC, y = Trait_PIC)) +

geom_point(color = "steelblue") +

geom_smooth(method = "lm", formula = y ~ 0 + x, se = FALSE, color = "black") +

facet_wrap(~ Metric, ncol = 3, scales = "free") +

geom_text(data = reg_data_snp, aes(x = Inf, y = Inf, label = label_slope),

hjust = 1.1, vjust = 1.5, inherit.aes = FALSE, size = 4.2) +

geom_text(data = reg_data_snp, aes(x = Inf, y = Inf, label = label_p),

hjust = 1.1, vjust = 3.0, inherit.aes = FALSE, size = 3.8) +

geom_text(data = reg_data_snp, aes(x = Inf, y = Inf, label = label_r2),

hjust = 1.1, vjust = 4.5, inherit.aes = FALSE, size = 3.6) +

labs(x = "Independent Contrasts: Number of Sympatric or Parapatric Congeners", y = "Independent Contrasts: Trait CV") +

theme_minimal(base_size = 12) +

theme(

panel.border = element_rect(color = "black", fill = NA, size = 0.8),

strip.background = element_rect(fill = "lightgray", color = "black"),

strip.text = element_text(face = "bold")

)

# === 5. PIC(S) vs trait PICs — regression through origin ===

plot_data_s <- pic_data %>%

pivot_longer(cols = all_of(facet_order),

names_to = "Metric", values_to = "Trait_PIC") %>%

mutate(S_PIC = rep(pic_data$S, times = length(facet_order)),

Metric = factor(Metric, levels = facet_order))

reg_data_s <- plot_data_s %>%

group_by(Metric) %>%

summarize(

slope = coef(lm(Trait_PIC ~ 0 + S_PIC))[1],

p = summary(lm(Trait_PIC ~ 0 + S_PIC))$coefficients[1, 4],

r2 = summary(lm(Trait_PIC ~ 0 + S_PIC))$r.squared,

.groups = "drop"

) %>%

mutate(

label_slope = paste0("slope = ", round(slope, 3)),

label_p = paste0("p = ", ifelse(p < 0.001, "< 0.001", round(p, 3))),

label_r2 = paste0("R² = ", round(r2, 3))

)

ggplot(plot_data_s, aes(x = S_PIC, y = Trait_PIC)) +

geom_point(color = "steelblue") +

geom_smooth(method = "lm", formula = y ~ 0 + x, se = FALSE, color = "black") +

facet_wrap(~ Metric, ncol = 3, scales = "free") +

geom_text(data = reg_data_s, aes(x = Inf, y = Inf, label = label_slope),

hjust = 1.1, vjust = 1.5, inherit.aes = FALSE, size = 4.2) +

geom_text(data = reg_data_s, aes(x = Inf, y = Inf, label = label_p),

hjust = 1.1, vjust = 3.0, inherit.aes = FALSE, size = 3.8) +

geom_text(data = reg_data_s, aes(x = Inf, y = Inf, label = label_r2),

hjust = 1.1, vjust = 4.5, inherit.aes = FALSE, size = 3.6) +

labs(x = "Independent Contrasts: Number of Sympatric Congeners", y = "Independent Contrasts: Trait CV") +

theme_minimal(base_size = 12) +

theme(

panel.border = element_rect(color = "black", fill = NA, size = 0.8),

strip.background = element_rect(fill = "lightgray", color = "black"),

strip.text = element_text(face = "bold")

)
